# Acceptable respondent burden in patient-reported outcome (PRO) assessment in oncology clinical trials: a mixed methods study with patients and trialists

**DOI:** 10.1186/s41687-026-00991-9

**Published:** 2026-02-19

**Authors:** Selin Yurdakul, Alexandros Georgiadis, Bert Sandy, Heiko Zettl, Brendon Wong, Anders Ingelgård

**Affiliations:** 1 IQVIA, London, England, U.K.; 2 Boehringer Ingelheim, Rhein, Germany

**Keywords:** Patient Reported Outcomes (PRO), Respondent burden, Clinical trial design, Outcomes research

## Abstract

**Background:**

Patient-reported outcomes (PROs) are essential for measuring the effectiveness of medical treatments from the patient’s perspective. PROs are typically defined as any measure that describes or reflects how a patient feels, functions, or survives, and are becoming increasingly important in regulatory decision making. The administration of PROs in clinical studies is often described as burdensome for both patients and trialists. This research aimed to examine the acceptable PRO burden from the perspective of patients and trialists, in the context of oncology clinical trials.

**Methods:**

This was a mixed-methods study, comprised of semi-structured qualitative interviews with oncology trialists and an online survey with people with cancer who reside in the United States, Canada, and Europe.

**Results:**

Nine trialists and 90 patients (M = 49.6 years old) with cancer took part in the study. Three overarching themes were identified across the collected data. The first theme describes the benefits of including PROs in oncology studies. The second theme describes the acceptable burden associated with PROs features, such as length, clarity, and relevance of items. The third theme describes implementation-related burden, such as frequency of assessment and use of ePROs.

**Conclusions:**

Both patients and trialists are willing to accept some of the burden associated with PRO administration in oncology clinical trials. Further research is needed to better understand how to best mitigate the challenges that increase respondent burden, over the threshold of acceptable burden within oncology clinical trials and beyond.

## Background

The United States (US) Food and Drug Administration (FDA) defines a patient reported outcome (PRO) as “any report of the status of a patient’s health condition that comes directly from the patient [or in some cases a caregiver or surrogate], without interpretation of the patient’s response by a clinician or anyone else” [1]. The FDA and the European Medicines Agency (EMA), have issued guidance documents to encourage sponsors to include PROs in drug development, including oncology, to amplify the patient voice [2]. However, the completion of PROs, particularly when requested regularly, can be burdensome for patients [3].

Aiyegbusi et al. [4] define respondent burden as “the degree to which a respondent perceives their participation in a task difficult, time consuming and emotionally stressful”. Factors that contribute to respondent burden, include the features of the chosen PRO (e.g., length, content, wording of items, and formatting), patient characteristics (e.g., general health status, literacy levels and cognitive status), mode of administration (e.g., electronic vs. pen and paper) and frequency of administration (e.g., weekly vs. daily) [4]. Some evidence suggests that there might be complex inter-relationships among these factors; for example, a recent-meta-analysis found that the length of a PRO may not be associated with respondent burden [5], and a study by Atkinson et al. found that patients with cancer reported minimal response burden even though they were asked to complete a large battery of PROs [6]. As such, sponsors may be puzzled with respect to how many PROs should be included in a clinical trial, how complex and lengthy they should be and how often patients should complete them.

PRO completion has a degree of burden on both patients and trialists; the first are asked to perform an activity (i.e., complete PROs), on top of numerous other study-related activities (i.e., blood tests, scans, medical examinations), and the second are asked to support the administration of PROs, on top of other administrative and clinical processes/procedures associated with the execution of an oncology clinical trial. From our perspective, what is important to understand, and has not been examined yet, is how much burden is acceptable when completing PROs. Understanding how much PRO-associated burden respondents are willing to accept as part of a clinical trial can provide valuable insights on how best to optimise their selection and implementation in clinical trials. This research aimed to examine the acceptable PRO burden from the perspective of patients and trialists, in the context of oncology clinical trials.

## Methodology

This was a multi-national, mixed-methods study, comprised of semi-structured qualitative interviews with Principal Investigators (PIs) and Clinical Trial Coordinators (CTIs), collectively referred to as trialists, to ensure we get a multi-perspective understanding of respondent burden from the trialist perspective within the oncology clinical trial context. In addition, an online survey was conducted with patients with cancer. This mixed-methods approach was chosen to help us elicit multi-dimensional and multi-perspective insights into how much PRO-related burden is acceptable in oncology clinical trials.

## Methods

### Recruitment

#### Patients with cancer

In 2022, patients with cancer were recruited through a US-based patient membership organisation. Individuals from the United States, United Kingdom, and Canada were invited via email to complete an online screener to determine eligibility (see Table 1) and then to complete the online survey. As our aim was to gain a global understanding of acceptable respondent burden within the oncology clinical trial context, we did not collect extensive medical information from participants.

#### Trialists

Purposive sampling was used to recruit trialists experienced with the administration of PROs as part of an oncology clinical trial. This method was selected to efficiently identify trialists who met the study eligibility criteria. The sponsor identified candidates from their network in the US, Canada, and Europe. Interested trialists completed an online screener, and if deemed eligible, they participated in a telephone interview.

**Table 1:** Eligibility criteria for study participation for patients and trialists

Eligibility criteria for patients with cancer	
**Inclusion criteria**	**Exclusion criteria**
Diagnosed with cancer	Not meeting the inclusion criteria
Completed a PRO(s) as part of an oncology clinical trial within the last 4 years	
Must reside either in the USA or Canada or the United Kingdom (UK)	
Able to read and understand English	
18 years old or older	
Must have access to an electronic device to complete the online survey	
**Eligibility criteria for Trialists**	
**Inclusion Criteria**	**Exclusion Criteria**
Have more than 5 years of experience in leading oncology clinical trials	Not meeting the inclusion criteria
Ability to understand and speak English fluently	
Have been involved in at least 3 oncology clinical trials that included scheduled PROs data collection (either pen-and-paper or ePROs)	
Willing to participate in a 1:1 telephone interview (lasting up to 60 min)	

## Data collection

A self-administered online survey questionnaire was developed to collect the views of people with cancer on their attitudes towards PRO administration. The survey included a combination of closed and open-ended questions, including free text responses to allow participants to provide further information and context to their responses. To ensure that the questions included in the survey were relevant and easy to understand, a PAG representative (who assisted with patient recruitment) reviewed the survey before its implementation.

A semi-structured discussion guide (see Table 2) was used to conduct the telephone interviews with trialists, which was moderated by one of the authors (SY). Verbal consent was obtained to audio record the interviews, and the transcripts were professionally transcribed verbatim. Before analysis, the transcripts were reviewed for data quality and anonymisation. Both the survey and the interview guide were purposefully developed to avoid any mention of the phrase “acceptable burden” or “PRO burden” to avoid biasing participants on reflecting PRO administration as burdensome from the outset. Instead, we chose to examine participants’ overall experience with PRO administration and allow different dimensions of acceptable or unacceptable PRO burden to surface spontaneously.

**Table 2:** Key topics and indicative line of questioning for trialist interviews

Topic	Indicative line of questioning
Trialist Background	-How many years of experience do you have as a site investigator? -What sorts of studies have you worked on (what fields)? -What type of institution is the site (academic, private, public)?
Attitudes towards PROs	-What is your overall attitude towards PROs in clinical trials? - Are there any challenges with including PROs in clinical trials? If yes, can you provide more details about each challenge?
Paper PROs	-What do you think about pen and paper versions of PROs? -Are there any benefits to the pen-and-paper mode of administration?
Electronic PROs	-What do you think about electronic versions of PROs? -Are there any benefits to an electronic mode of administration?
Electronic Devices	Have you worked on any studies where patients had the option to use an app on their personal phones? -If YES: What was that experience like? Similar or different from other ePRO or paper studies? -If NO: Do you think that would be a good solution for patients
At-home PROs	-What issue could be experienced when completing PROs at-home ? -What are the benefits of completing PROs at-home?
On-site PROs	-What are the benefits of completing PROs on site?
Research staff	-Do you feel like you have enough employees to efficiently administer PROs?
Training for Trialists	-How kind of training do you/clinical site staff receive on administering PROs? -How do you communicate the importance of completing all PROs to patients and clinical site staff?

### Analysis

#### Survey data

The survey data were analysed using Microsoft Excel, with separate tabs used to organize different sections of the survey, including key socio-demographic and clinical information (i.e., cancer types) and participants’ responses to the survey questions. Most of the survey consisted of closed-ended questions, for which frequency tables were generated to summarize the number of participants selecting each response option. Some of these closed-ended questions were followed by open-ended prompts, inviting participants to elaborate on their responses. The open-ended texts from the relevant questions were grouped and reviewed according to the corresponding response options to provide deeper insight into participants’ reasoning. For example, responses from participants who selected electronic PROs as their preferred method of completion were clustered to examine the motivations behind their preference, adding depth and context to the quantitative findings.

#### Semi- structured interview data

The qualitative data were analysed using Microsoft Excel. Separate tabs were created for each interview topic such as professional background, attitudes toward PROs, and preferences for paper versus electronic methods. Each trialist was assigned a column, with interview questions listed in rows. Participants’ responses were entered alongside each question to enable side-by-side comparison. Summaries were added to each response to capture key themes and insights across participants.

Both the interview and survey data were analysed by SY using a thematic analytic approach that combined inductive and deductive coding approaches to summarise data into overarching categories and themes [7].to enhance the validity and depth of the findings, the team systematically compared and cross-referenced patterns and insights that emerged from the quantitative survey responses and the qualitative interview data, particularly in relation to the concept of acceptable PRO burden. Themes identified through the thematic analysis of the interview data were refined by examining how they explained survey findings and vice versa.

## Results

### Survey participants

In total 90 participants completed the online survey. The mean age of the participants was 49.67 years (range: 24–77 years). The sample was composed of 60 females (*n* = 60, 67%), 26 males (*n* = 26, 29%), 2 genderqueer (*n* = 2, 2%), 1 non-binary (*n* = 1, 1%) and 1 participant (*n* = 1, 1%) did not disclose their gender (Table 3) Over 90% of the sample, reported that they had been diagnosed with a solid tumor.

**Table 3:** Socio-demographic and clinical characteristics of survey participants

Socio-demographic and clinical characteristics	*N* (%)
**Age**	
Mean	49.67
Median	48.50
Age range	24–77
**Country of Origin**	
USA	83 (92%)
Canada	4 (4%)
UK	3 (3%)
**Gender**	
Female	60 (67%)
Male	26 (29%)
Genderqueer	2 (2%)
Non-binary	1 (1%)
I prefer not to say	1 (1%)
**Ethnicity**	
White alone non-Hispanic	67 (74%)
Black or African alone non-Hispanic	5 (6%)
Asian alone non-Hispanic	4 (4%)
English, Welsh, Scottish, Northern Irish or British	4 (4%)
Hispanic or Latino	3 (3%)
Multiracial non-Hispanic	3 (3%)
Blank	3 (3%)
Other race alone non-Hispanic	1 (1%)
**Employment status**	
Full-time employment	25 (28%)
Unemployed due to long-term sickness	25 (28%)
Part-time employment	20 (22%)
Other	17 (19%)
Unemployed	2 (2%)
**Marital Status**	
Married	52 (58%)
Single	20 (22%)
Divorced	13 (14%)
Separated	4 (4%)
Widowed	1 (1%)
**Type of Cancer**	
Solid tumors	80 (89%)
Hematological cancers	10 (11%)

### Interviews with trialists

In total of nine interviews were conducted with trialists across the selected countries. Participant demographics are presented in Table 4.

**Table 4:** Demographic characteristics and experience of trialists

Demographic	*N* (%)
**Profession**	
Principle Investigator/Site Investigator	6 (67%)
Clinical Trial Coordinator	3 (33%)
**Years of experience in oncology clinical trials**	
Less than 10 years	3 (33%)
Between 10 to 20 years	5 (56%)
More than 20 years	1 (11%)
**Location**	
United Kingdom	5 (56%)
USA	3 (33%)
Canada	1 (11%)
**Type of Institution**	
Academic Institution/Public Hospital	6 (66%)
Private Hospital	3 (33%)
**Experience with Pen & Paper or ePROs**	
Yes, both	8 (88%)
Only paper and pen	1 (11%)
Only ePROs	0 (0%)
**Experience with a wearable device**	
Yes	4 (44%)
No	5 (56%)

### Findings

The thematic analysis process identified the following three themes: “Benefits of including PROs in oncology studies”, “Burden due to PROs features” and “Implementation-related burden”.

### Benefits of including PROs in oncology studies

Trialists and survey participants reported positive attitudes towards PROs and described them as an important method to communicate the patients’ voice in oncology drug development. All 9 trialists unanimously reported that the inclusion of PROs in clinical trials is important because they can provide more context to the clinical data and can capture dimensions of patient experience that cannot be measured by traditional methods (i.e., biomarkers).

*They can help putting in context*,* for instance*,* the efficacy of novel drugs or the toxicity of novel drugs*,* because they capture a different dimension. They capture how both the intensity of the treatment*,* for instance*,* how the side effects of the treatment*,* how the efficacy of the treatment translates into the patient’s experience more directly. Overall*,* the impression is positive. They’re important. (Principal Investigator)*

The trialists also mentioned the growing recognition of PRO data from regulatory agencies and the necessity of incorporating PROs into clinical trials.

*It’s increasingly commonplace to incorporate PROs within clinical trials. I guess the regulators*,* of course*,* are increasingly demanding of good-quality data in terms of patient-reported outcomes and quality of life. (Clinical trial coordinator)*

Of the 90 survey participants, 92% (*n* = 83) either ‘strongly agreed’ or ‘agreed’ that PROs can help them express how they felt and functioned while in an oncology clinical trial. Similarly, 92% (*n* = 83) of participants either ‘strongly agreed or ‘agreed’ that PRO questionnaires are important to demonstrate the patient perspective, and they should be included as standard in clinical trials. (Table 5)

**Table 5:** Survey participant responses to statements on the value of PROs in oncology trials

Survey questions	Strongly agree *N* (%)	Agree *N* (%)	Disagree *N* (%)	Strongly disagree *N* (%)	I don’t know or have an opinion *N* (%)
PRO questionnaires can help me express how I feel and function whilst participating in a clinical trial	46 (51%)	37 (41%)	5 (6%)	0 (0%)	2 (2%)
PRO questionnaires are important to demonstrate the patient perspective, and they should be included as standard in clinical trials	54 (60%)	29 (32%)	2 (2%)	0 (0%)	5 (5%)

### Burden due to PRO features

This theme describes the burden associated with the sub-optimal design and selection of PROs considering the needs of patients with cancer.

### Length of the PROs

Across the collected data both interviews and surveys indicated that lengthy PROs, meaning PROs that have a high number of questions and/or take too long to complete, were described as burdensome, contributing to patient disengagement and missing data. One trialist specifically identified lengthy PROs as the primary cause of missing data in their experience.

*I think from a patient’s perspective*,* the length of the questionnaire*,* the number of questions*,* the number of questionnaires*,* because some studies have 5 or 6 that are required can lead to them not completing the questionnaires. I think I would say that’s #1 (Clinical trial coordinator)*

More than half of the survey participants reported that they either ‘strongly agreed’ or ‘agreed’ that answering too many questions could discourage them from completing PROs in the future. Most of the participants also indicated that having sufficient time to complete the PROs was important, suggesting that both the length of the questionnaire and the time allocated for completion are critical factors influencing perceived PRO burden (Table 6).

**Table 6:** Survey participant responses to statements on PROs questionnaire length and time allocated for completion

Survey questions	Strongly agree *N* (%)	Agree *N* (%)	Disagree *N* (%)	Strongly disagree *N* (%)	I don’t know or have an opinion *N* (%)
Having to answer too many questions can affect my willingness to complete PRO questionnaire(s) in the future	10 (11%)	35 (39%)	26 (29%)	13 (14%)	6 (7%)
It is important to have enough time to complete PROs in the future	40 (44%)	46 (51%)	1 (1%)	0 (0%)	3 (3%)

Survey participants also indicated their preferred duration for completing PROs at a given time. Of the 90 survey participants 38% (*n* = 34), reported that PROs should not take more than ‘5–10 minutes’ to complete, followed by 30% (*n* = 27), who reported no longer than ‘11-15minutes’. More than half of the survey participants 68% (*n* = 61) reported that PROs should not take longer than ‘5 to 15 minutes’ to complete. One participant noted that the acceptable duration may depend on the frequency of administration, suggesting that a longer time frame may be acceptable if the PRO is administered only once (Table 7).

**Table 7:** Survey participant preferences for time allocated to complete PROs questionnaires

How long it should take to complete PROs in a clinical trial?		
Survey Response Options	No. of Responses from 90 Participants	Indicative quotes from survey participants
5–10 min N (%)	34 (38%)	*“I think attention spans for those of us going through illness or stress are far shorter. You’ll get MUCH better answers and engagement with short*,* to-the-point questions.” (Survey participant)* *“Over 10 minutes is too long for a PRO. Adding a comment section would be useful for patients to add additional information if needed.” (Survey participant)* *“I would be willing to regularly provide 11–15 minutes to a study questionnaire. A one-off questionnaire could be more than 25 minutes. I think less than 10 might not be enough time to fully capture the details of the patient experience.” (Survey participant)*
11–15 min N (%)	27 (30%)	
16–20 min N (%)	16 (18%)	
21–25 min N (%)	4 (4%)	
Over 25 min N (%)	1 (1%)	
I don’t know or have an opinion N (%)	8 (9%)	

### Questions difficult to understand

Across the data, it was evident that the inclusion of questions in PROs that are difficult to understand was considered a major burden by both survey participants and trialists. One trialist noted that this was the primary factor discouraging engagement with PROs.

*Probably the complexity of the questions themselves*,* if I’m honest. That’s*,* in my view*,* factor #1 (Principal Investigator)*

Most of the survey participants 82% (*n* = 74) either ‘strongly agreed’ or ‘agreed’ with the statement *“Difficult words to understand should be kept at a minimum in PRO questionnaires”*, and 81% (*n* = 73) of the participants either strongly agreed or agreed with the statement *“I am less likely to answer a question included in a PRO questionnaire if I do not understand*”. Finally, 77% (*n* = 70) of the survey participants either ‘strongly agreed’ or ‘agreed’ with the statement “*I am less likely to answer a question included in a PRO questionnaire if the wording is unclear​”* (Table 8).

**Table 8:** Survey participant responses to statements on the clarity of PRO questionnaire items

Survey questions	Strongly agree *N* (%)	Agree *N* (%)	Disagree *N* (%)	Strongly disagree *N* (%)	I don’t know or have an opinion *N* (%)
Difficult words to understand should be kept at a minimum in PRO questionnaires	32 (36%)	42 (47%)	7 (8%)	2 (2%)	7 (8%)
I am less likely to answer a question included in a PRO questionnaire if I do not understand	24 (27%)	49 (54%)	10 (11%)	3 (3%)	4 (4%)
I am less likely to answer a question included in a PRO questionnaire if the wording is unclear”.	18 (20%)	52 (58%)	12 (13%)	3 (3%)	5 (6%)

### Irrelevant questions

The relevance of the questionnaire refers to the extent to which respondents perceive the questions included in the PRO relevant to their experience. Trialists also commented that patients often report that having to answer questions that are not important to them is particularly burdensome.

*Well*,* I guess there is a potential that for some of these more generic questionnaires*,* which by their very definition they tend to be…they’re trying to capture broad themes rather than specific things… They may be a bit disillusioned by the questionnaire in that it’s not really representing them. I’ve heard a couple of patients say that. That they’re asking me about all the things that are fine*,* but they don’t ask me about the things that aren’t fine. (Principal Investigator)*

Most of the survey participants, 88% (*n* = 79), either ‘strongly agreed’ or ‘agreed’ that they are more likely to complete a PRO that includes questions that are specific/relevant to their health condition (Table 9).

**Table 9:** Survey participant responses to statements on the importance of PRO questionnaire item relevance

Survey questions	Strongly agree *N* (%)	Agree *N* (%)	Disagree *N* (%)	Strongly disagree *N* (%)	I don’t know or have an opinion *N* (%)
I am more likely to complete a PRO questionnaire that includes questions that are specific and relevant to my health condition and treatment than to complete a questionnaire that does not include these questions	44 (49%)	35 (39%)	8 (9%)	2 (2%)	1 (1%)

### Implementation-related burden

This theme describes the factors that contribute to respondent burden and are associated with the implementation of PROs in clinical trials, including frequency of PRO administration, challenges burden associated with clinical sites, and technical difficulties with ePROs.

The frequency of PRO administration can contribute to respondent burden. One trialist reported that frequent PRO administration can be burdensome for both patients and trialists, particularly when ensuring adherence to protocol timelines.

*When are these PROMs being measured? Are they monthly*,* every 3 months*,* every 6 months*,* so the schedule of those both in terms of the frequency of those potentially adding to sort of additional work for both the patient and the site personnel staff. Then actually remembering to make sure that they are being completed at the correct time*,* so in line with the protocol* Principal Investigator

The majority 54%, (*n* = 49) of the survey participants expressed willingness to complete PROs either weekly or monthly. Additionally, 23% (*n* = 21) indicated they would be comfortable completing them as often as needed. Conversely, very few patients were willing to complete PROs twice daily or only every six months (Table 10).

**Table 10:** Survey participant preferences on the frequency of PRO questionnaire administration

How often you would be willing to complete a PRO questionnaire?		
Survey Response Options	No. of Responses from 90 Participants	Indicative quotes from survey participants
Twice per day N (%)	1 (1%)	*“I wouldn’t want to complete PROs every day or weekly indefinitely*,* but monthly is a reasonable amount of time in my opinion to schedule to fully complete a questionnaire. Anything more*,* I may not have the time to answer completely or fully.” (Survey participant)*
Once per day N (%)	17 (19%)	
Once per week N (%)	24 (27%)	
Once per month N (%)	24 (27%)	
Once every six months N (%)	3 (3%)	
As often as necessary N (%)	21 (23%)	

More than half of the survey participants, 53% (*n* = 48) reported that they would prefer to complete PROs at home, whereas only, 33% (*n* = 30) reported that they would prefer to complete them at the clinic. An additional 10% did not express a specific preference for either setting. Home administration was preferred because of the benefits it offers, including convenience and privacy, it was considered less intimidating and easier for participants to keep focus on PRO completion (Table 11).

**Table 11:** Survey participants’ response to their preferred location to complete the PROs

What is your preferred location to complete a PRO questionnaire?		
Survey Response Options	No. of Responses from 90 Participants	Indicative quotes from survey participants
Home: N (%)	48 (53%)	“*It seems it is less pressure if you complete them at home.”* *“More comfortable at home and for cancer patients the less they can be in the hospital the better.”* *“It is less distracting to complete questionnaires at home.”* *“I feel rushed when I am filling out questions when others are around. So if people are finishing quickly then I think I must be too slow and being judged. My answers are more accurate.”* *“I’m in my own sanctuary*,* away from clinic/hospital setting that’s already intimidating.”*
Clinic: N (%)	30 (33%)	
Home or Clinic: N (%)	9 (10%)	
Other: N (%)	2 (2%)	
No preference: N (%)	1 (1%)	

Survey participants emphasized the importance of support from site staff when completing PROs. A total of 88% (*n* = 79) agreed that site staff should encourage patients to complete any missed questions. Similarly, 87% (*n* = 78) reported that patients should receive guidance from site staff on how to complete PROs. Clear and comprehensive explanations from site staff were also valued: 87% (*n* = 78) participants reported being more willing to complete PROs when they understood why they were being asked to do so, and 82% (*n* = 74).

### Trialists reported similar views

*In fact*,* probably the single most important determinant of completion rates is really the attitude and the support given to the patient by those who are helping to look after the patient. I think if you want to maintain completion rates*,* that’s one of the main areas to look at. (Principal Investigator)*

Trialists also talked about the challenges associated with retaining staff to provide support to patients completing PROs:

*The perception is*,* and the reality is now increasingly that we struggle to maintain our teams. We have*,* I think*,* across the health service*,* and this is true in our center as well as elsewhere. I think it’s true across Europe and the rest of the world*,* in fact. (Principal Investigator)*

Most trialists described ePROs as an efficient solution to collect PRO data and to ensure data integrity. However, most of them reported concerns with technical issues and device functionality. Technical barriers, such as IT issues (e.g., poor Wi-Fi connection, web browser incompatibility) and data security of patient data were some of the reasons cited about why patients do not complete ePROs. One of the trialists commented:

*I think that the big thing you hear about is the devices*,* whether they’re working or not. I think the patients don’t find it overly bothersome. I think they feel like it kind of work for the study in a way. But I think that the nurses and the kind of support staff do find it challenging sometimes because they spend hours sometimes on hold with…(Principal Investigator)*

Similarly, most of the survey participants 58% (*n* = 52) reported that they preferred ePROs to pen-and-paper PROs. However, a notable proportion, 32% (*n* = 29) reported that technical difficulties made the use of ePROs burdensome. Problems with charging the device, connectivity issues, browser compatibility issues, and login errors were some of the technical problems that survey participants reported.

Seven trialists, expressed concerns about the difficulties faced by elderly patients who may have less familiarity and confidence using electronic devices.

*The only disadvantage there is that if you’re using technology*,* then older patients*,* particularly patients with cancer*,* may be less…less confident to use technology. Family members may be a dependency. (Principal Investigator)*

## Discussion

This study aimed to examine the burden patients would be willing to accept when completing PROs as part of an oncology clinical trial. To our knowledge, this is the first study that compared the perception of PRO burden between people with cancer and oncology trialists, aiming to examine how each group understands acceptable PRO respondent burden. Our findings revealed strong positive attitudes towards PROs among both trialists and patients with cancer, confirming the important role that PROs play in amplifying the patient voice in oncology drug development. The factors contributing to respondent burden were broadly similar between both groups and included administration of lengthy, difficult to understand and irrelevant PROs, high frequency of PRO administration, and technical difficulties with electronic PRO administration. Most of these factors have already been mentioned in the previous literature and our findings are broadly consistent with them [8–11].

What our research adds to the respondent burden literature is the degree over which these factors become burdensome and may lead to poor completion rates, missing PRO data or trial participant withdrawal. Both people with cancer and trialists unanimously reported that it is unacceptable to administer PROs with content that is irrelevant to the disease or content that is difficult to understand. Similarly, for both groups it was largely unacceptable to select lengthy and complicated PROs, especially in a patient population that often experiences high levels of fatigue, hence, they might not have the cognitive and physical capacity to complete them. Most participants reported that it was acceptable to spend up to 15 min to complete PROs, with only a few of them increasing the acceptability threshold to up to 20 min. For many participants, having to spend over 20 min to complete PROs was an unacceptable burden that was very likely to deter them from completing PROs, especially if they have to complete them regularly, which is often the case in oncology clinical trials. These findings are at odds with the findings reported by Atkinson TM, et al. [6] who found that patients reported minimal burden when they had to complete a long battery of PROs. Such findings demonstrate the complexity that characterises the inter-relationships among the factors that may lead to respondent burden.

ePROs were praised for the convenience they offered to patients and trialists alike, as patients can complete them at their convenience, outside of clinic visits. However, the analysis of both the interview and the survey data revealed that technical issues associated with either the device used to complete the PROs or the ePROs themselves contribute to respondent burden. Despite such technical challenges, most of the survey participants favoured ePROs for the flexibility they offer to complete them outside of a clinic visit.

PROs are increasingly becoming an essential component of the drug development process. In oncology, PROs are considered an integral part of the clinical development programs of new drugs, therefore, ensuring that the selected PROs are fit-for-purpose is of utmost importance. Many of the “unacceptable burdens” identified in this study can be mitigated with early planning and close adherence to regulatory guidance, such as the series published by the FDA on Patient Focused Drug Development (PFDD) (17–20). Recently, Aiyegbusi et al. [4] published a set of recommendations on how to address respondent burden associated with PRO assessment. The authors developed 19 recommendations focusing on three distinct phases of PRO selection and implementation, including (1) rationale and schedule for PRO assessment, (2) measure selection and (3) measure delivery. Most of these recommendations are closely aligned with the findings of this study that demonstrate their relevance to patients participating in oncology clinical trials. Additionally, sponsors should aim to engage with patients early, understand what matters to them the most and why, and measure these aspects using validated PROs. The science of PRO development and validation is evolving, with techniques such as computer adaptive testing, item response theory, skip sequencing, and item banking potentially decreasing respondent burden compared to pen and paper forms.

This study is not without limitations. First, we did not collect detailed information on the treatment history of the survey participants (e.g., type of treatment received, duration of treatment). These factors could have influenced participants’ experience completing PROs, and therefore, their attitudes towards them. Second, based on the close-ended questions responses, survey participants did not provide detailed accounts of what would be considered acceptable PRO burden. Lastly, the survey results can be affected by respondent motivation, availability, and willingness and this may have affected participants’ responses to certain questions.

Further research is needed to better understand and define acceptable respondent burden within the context of oncology clinical trials, and beyond. Qualitative interviews with patients could provide deeper and more comprehensive insights into defining what is an acceptable PRO-burden considering the multitude of procedures that patients have to follow when taking part in clinical trials. Additionally, further investigation is needed to better understand the challenges of PRO administration from the trialist perspective, how these challenges may contribute to the respondent burden, as well as how these challenges may differ between industry sponsored and academic/healthcare sponsored clinical studies.

## Data Availability

The datasets used and/or analysed during the current study are available from the corresponding author on reasonable request.

## Abbreviations

BYOD: Bring Your Own Device
ePROs: electronic Patient Reported Outcomes
EMA: European Medicines Agency
FDA: Food and Drug Administration
PFDD: Patient Focused Drug Development
PROs: Patient-reported outcomes
UK: United Kingdom
US: United States
